# Incidence, outcome, and prognostic factors of prolonged mechanical ventilation among children in Chinese mainland: a multi-center survey

**DOI:** 10.3389/fped.2024.1413094

**Published:** 2024-05-30

**Authors:** Zhengzheng Zhang, Xiaodi Cai, Meixiu Ming, Li Huang, Chengjun Liu, Hong Ren, Dong Qu, Hengmiao Gao, Yibing Cheng, Furong Zhang, Zihao Yang, Wei Xu, Hongjun Miao, Pan Liu, Yuxin Liu, Guoping Lu, Weiming Chen

**Affiliations:** ^1^ Pediatric Intensive Care Unit, National Children’s Medical Center, Children’s Hospital of Fudan University, Shanghai, China; ^2^ Department of Pediatric Intensive Care Unit, National Children’s Medical Center for South Central Region, Guangzhou Women and Children’s Medical Center, Guangzhou, China; ^3^ Department of Pediatric Intensive Care Unit, Western Pediatric Development Union, Children’s Hospital of Chongqing Medical University, Chongqing, China; ^4^ Department of Pediatric Intensive Care Unit, National Children’s Medical Center, Shanghai Children’s Medical Center, Shanghai Jiaotong University School of Medicine, Shanghai, China; ^5^ Department of Pediatric Intensive Care Unit, Children’s Hospital, Capital Institute of Pediatrics, Beijing, China; ^6^ Department of Pediatric Intensive Care Unit, National Center for Children’s Health, Beijing Children’s Hospital, Capital Medical University, Beijing, China; ^7^ Department of Pediatric Intensive Care Unit, Children’s Hospital Affiliated to Zhengzhou University, Zhengzhou, China; ^8^ Department of Pediatric Intensive Care Unit, Wuhan Children’s Hospital, Tongji Medical College, Huazhong University of Science & Technology, Wuhan, Hubei, China; ^9^ Department of Pediatric Intensive Care Unit, National Clinical Research Center for Child Health, Children’s Hospital of Zhejiang University School of Medicine, Hangzhou, Zhejiang, China; ^10^ Department of Pediatric Intensive Care Unit, National Children’s (Northeast) Regional Medical Center, Shengjing Hospital of China Medical University, Shenyang, China; ^11^ Department of Emergency/Critical Medicine, Children’s Hospital of Nanjing Medical University, Nanjing, Jiangsu, China

**Keywords:** prolonged mechanical ventilation, prognostic factors, pediatric intensive care unit, critical care, chronic respiratory failure

## Abstract

**Objective:**

To evaluate the incidence, outcome, and prognostic factors of prolonged mechanical ventilation (PMV) in children in Mainland China.

**Methods:**

A prospective study was conducted in 11 pediatric intensive care units (PICUs) from May 1, 2021, to April 30, 2022. All pediatric patients on mechanical ventilation meeting the criteria for PMV were included in the study.

**Results:**

Out of 5,292 patients receiving mechanical ventilation, 278 children met the criteria for PMV (5.3%). After excluding case with incomplete data or lost to follow-up, the study included 250 patients. Among them, 115 were successfully weaned from mechanical ventilation, 90 died, and 45 were still on mechanical ventilation. The 6-month survival rate was 64%. The primary associated conditions of PMV were lower airway diseases (36%), central nervous system diseases (32%), and neuromuscular diseases (14%). The stepwise multiple logistic regression analysis indicated that the utilization of vasoactive agents and an elevated pediatric logistic organ dysfunction-2 (PELOD-2) score on the day of PMV diagnosis were significantly associated with an increased of PMV death. Specifically, the odds ratio (OR) for vasoactive agent use was 2.86; (95% CI: 0.15–0.84; *P* = 0.018), and for the PELOD-2 score, it was 1.37; 95% CI: 1.17–1.61; *P* < .001). Conversely, early rehabilitation intervention was negatively associated with the risk of PMV death (OR = 0.45; 95% CI: 0.22–0.93; *P* = .032). Furthermore, the tracheotomy timing emerged as an independent predictor of failure to wean from PMV, with an OR of 1.08, (95% CI: 1.01–1.16; *P* *=* .030).

**Conclusions:**

The study revealed a 5.3% incidence of PMV in children requiring mechanical ventilation in China. The use of vasoactive agents and a higher PELOD-2 score at PMV diagnosis were significantly associated with an increased risk of PMV death, whereas early rehabilitation intervention was identified as crucial for improving patient outcomes. The timing of tracheostomy was identified as a high-risk factor for failure to wean from mechanical ventilation.

## 1 Introduction

As more pediatric intensive care unit (PICU) patients survive, the incidence of long-term complications is escalating, resulting in a growing population of patients transitioning to a chronic stage (1, 2). Individuals in this stage often necessitate ongoing life-sustaining interventions, including mechanical ventilation (MV). Opting out of prolonged mechanical ventilation (PMV) is associated with an almost 100% mortality rate. Therefore, despite the substantial risks associated with PMV, it remains the sole viable option for parents who are not yet ready to transition to palliative or comfort care. Studies in adult have reported that the incidence of PMV ranges from 4% to 13% in patients requiring ventilation, yet they consume 40% of critical care medical resources (3). Among pediatric patients, reports have indicated that 4%–7% ventilated patients required PMV, and the number of children requiring PMV has been rapidly increasing in recent years (4–7).

PMV is associated with high mortality (1, 8). In the United States, the first-year mortality rate of children requiring PMV was 73% (9), while in Taiwan in 2010, the mortality rate was 45% (10). In a systematic review examined risk factors for PMV and weaning failure (11). Out of 532 articles identified, 23 studies with a total of 23,418 patients were included. Risk factors identified as relevant for PMV or prolonged weaning include age, comorbidities (such as previous stroke, renal impairment, poor cardiac function, chronic obstructive pulmonary disease), and various laboratory parameters (such as low platelets, elevated blood urea nitrogen, elevated creatinine, low serum albumin, elevated blood glucose levels or hypernatremia). The authors noted that a direct comparison of risk factors was challenging due to the heterogeneity of the studies. Multidimensional scores were more likely to reflect the full spectrum of patients ventilated in different ICUs than single risk factors.

Due to the lack of PMV management facilities in mainland China, such as Long-Term Acute Care Hospitals (LTAC), nursing home, and Ventilator Rehabilitation Unit (VRU), children requiring PMV often remain in the PICU for extended periods, leading to high mortality rates and a heavy disease burden. To strengthen PMV management, following the example of the United States and Canada's management of home mechanical ventilation (HMV), we established the PMV Collaboration Group in 2021 and created a standardized database for all PMV patients admitted to PICUs across China. Data was collected from a total of 11 tertiary hospital in 9 provinces and municipalities across China, including two national children's medical centers, four regional children's transferring centers and five provincial children's medical centers.

Preliminary research indicated that central nervous system and neuromuscular diseases were more likely to cause PMV (12). Risk factors influencing PMV, in addition to the underlying diseases, were associated with the child's age (<1 year old), a higher Pediatric Index of Mortality 3 score (PIM 3) at admission, prematurity, the use of inotropes or vasopressors, extubating failure, and ventilator mode on the first day of MV. Furthermore, the complications arising from ventilator dependence significantly contribute to mortality. Therefore, further investigate into high-risk factors for mortality and weaning failure is crucial. This multi-center study followed patients for at least 6 months with the objective of describing the incidence, short-term outcome, and prognostic factors of PMV among ventilated children in PICUs in Mainland China.

## 2 Methods

### 2.1 Study design and settings

This prospective multi-center cohort study was conducted by the PMV Collaboration Group led by Children's Hospital of Fudan University. The PMV Collaboration Group is a national collaborative research network focusing on PMV clinical studies in China, including major cities such as Beijing, Shanghai, Guangzhou, Chongqing, Zhejiang, Jiangsu, Zhengzhou, Wuhan and others, ensuring a representative population sample.

The study was reviewed and approved by the Institutional Review Board (IRB) of Children's Hospital of Fudan University with the file number 2020-475 and the study was registered on Clinicaltrial.gov with the identifier NCT04511741.

Guardians of all participants were informed and signed a routine admission consent form which included consent for the future use of their data for research purposes. No specific consent form for this particular study was signed by the guardians.

### 2.2 Study population

Inclusion Criteria: All patients aged between 28 days and 18 years who met the criteria for PMV and were admitted to participating PICUs between May 1, 2021, and April 30, 2022 were included in this study. PMV was defined (13, 14) as the requirement for invasive and noninvasive mechanical ventilation for a duration of 21 consecutive days or more, with ventilation exceeding 6 h per day. Exclusion Criteria: Children eligible for inclusion in this study are those who have recently received mechanical ventilation, with PMV being diagnosed after admission to the PICU. However, children who had previously undergone mechanical ventilation before PICU admission with an unclear duration, or those who had been ventilated for over 21 days, are excluded from the study. Follow-Up: Following discharge, all participants were followed up for a period of 6 months through telephone interviews or outpatient visits.

### 2.3 Data collection and outcomes

Each participating center implemented standardized lung-protective ventilation strategies and protocols to prevent ventilator-associated pneumonia (VAP) (15) in children receiving mechanical ventilation. Sedative and analgesic agents for pediatric patients were administered in accordance with the Chinese guidelines for children's sedation and analgesia (16). A standardized protocol for comfort-oriented, minimal sedation was routinely implemented, with the goal of sustaining a minimal level of sedation whenever practicable. The depth of sedation was systematically evaluated through comfort scoring instruments and daily arousal assessments. In the absence of evidence-based guidelines for safely weaning sedation in patients undergoing PMV, PICU teams generally commence the weaning process as soon as the patient's clinical condition permits, ensuring sedation is used only when vital for maintaining clinical stability. The diagnosis of VAP and catheter related infection followed the criteria outlined in the 2001 Hospital Infection Diagnostic Standard of the Ministry of Health (17). Children requiring PMV received early initiation of enteral nutrition and rehabilitation. Home mechanical ventilation were recommended for all PMV patients who met the discharge criteria. Family caregivers underwent training on essential skills, including cardiopulmonary resuscitation, airway management, and ventilator operation prior to patient discharge.

Each research center appointed a part-time research assistant who underwent standardized training to enroll PMV cases and gather clinical information. Baseline data, collected on day 0 (defined as the PMV diagnosis date), including age, gender, height, weight, underlying associated conditions of PMV, Pediatric Logistic Organ Dysfunction-2 (PELOD-2) score, types of mechanical ventilation, antibiotic and vasoactive agents’ usage (referred to dopamine, dobutamine, milrinone, epinephrine, and norepinephrine), rehabilitation interventions, nutrition status and laboratory findings. Subsequent clinical data were collected on days 14, 28, 60, 90, and 180, including encompassing duration of ventilation, extubation/tracheotomy dates, complications, ICU length of stay, and mortality.

The study endpoint was set at 180 days or death, whichever came first. The primary outcomes focused on PMV cases mortality, as well as identifying risk factors associated with death and successful ventilator weaning.

### 2.4 Definitions

The diagnostic criteria for VAP (18) were as follows: the presence of a new and persistent (for at least 48 h) or progressive radiographic infiltrate, coupled with at least one of the following: fever >38.4°C or <36°C, a blood leukocyte count of >15,000 mm^3^ or <4,000 mm^3^.

Early rehabilitation was defined as the initiation of rehabilitation activities once the patient's condition stabilized, typically commencing within 3 days after intubation. The rehabilitation process was expertly guided by a skilled rehabilitation physician. The primary components of the rehabilitation program encompassed both active and passive limb movements, respiratory muscle training, and electrical muscle stimulation. Passive limb exercises included body posturing, positioning limbs in functional positions, and enhancing joint mobility. Active limb exercises incorporated a range of activities such as turning over in bed, transitioning from bed to sitting, transferring from bed to chair, standing, assisted walking, independent walking, and strengthening trunk control and muscles at the bedside. Respiratory muscle training focused on manual diaphragm exercises, which involved abdominal breathing, diaphragmatic stretching, manual diaphragm release, and external diaphragm electrical stimulation. The top three exercises were each conducted for 10 min, totaling 30 min per session, and were performed twice daily. All centers were equipped to administer these exercises. External diaphragm electrical stimulation utilized an external diaphragmatic pacemaker to stimulate the diaphragmatic nerve via a body surface electrode, thereby enhancing diaphragmatic contraction function. This stimulation was applied for 15 min each time, twice a day. The aforementioned rehabilitation exercises were not reliant on any specialized facilities, with the exception of the external diaphragmatic pacemaker, which was available at only two centers.

### 2.5 Statistical analysis

The statistical analyses were carried out using STATA 14.0 (Stata Statistical Software, Stata Corp, College Station, TX). Given the non-normal distribution of variables, the descriptive characteristics were reported as medians and interquartile rang (IQR) for continuous variables, and as number and relative frequency for categorical variables. The Wilcoxon rank-sum test and Pearson chi-squared test were employed for comparing continuous and categorical variables between the survivors and non-survivors' groups, as well as between the successful weaning and failure to wean groups, respectively. A multiple logistic regression analysis was performed on all risk factors related to mortality and failure to wean in patients requiring PMV. Stepwise logistic regression, which involves backward elimination of factors at a significance level of *p* > 0.1, was chosen for this analysis. Significant risk factors were presented with odds ratios (OR) and 95% confidence intervals (CI). A significance level of *P* < 0.05 was considered statistically significant for all analysis.

## 3 Results

### 3.1 Incidence and outcomes of children with PMV

**Figure 1:**
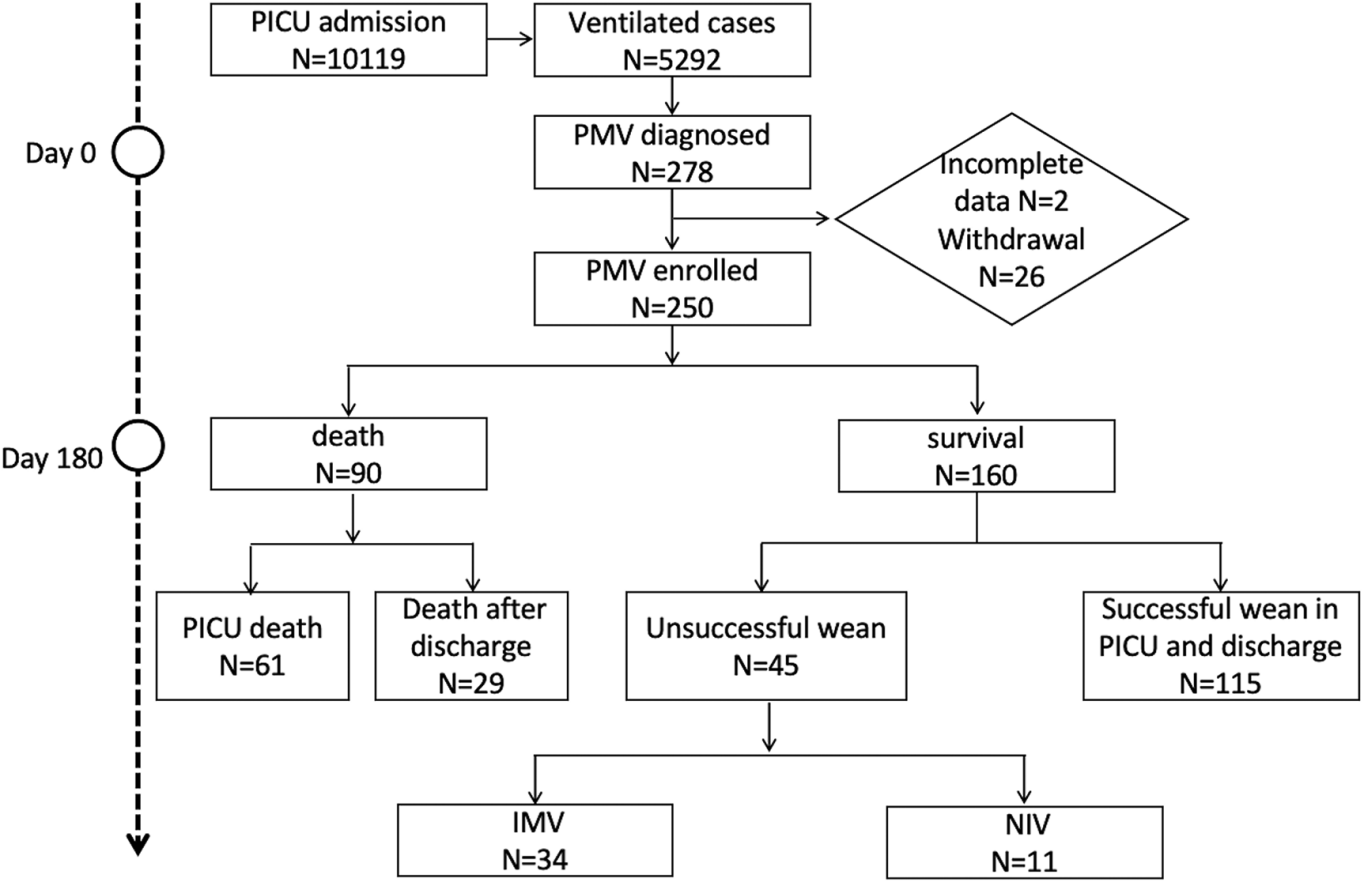
Flowchart. PICU, Pediatric Intensive Care Unit; IMV, Invasive Mechanical Ventilation; NIV, Non-Invasive Mechanical Ventilation.

During the study, 5,292 patients received mechanical ventilation in 11 PICUs. Among them, 278 children (5.3%) needed PMV. After excluding incomplete data and lost follow ups, a total of 250 patients were analyzed (Figure 1). At the 180-day follow-up, 115 patients (46%) successfully weaned off ventilation and discharged from the PICU, while 90 patients (36%) died. Among the deceased, 61 died in the PICU, and 29 families decided to stop their children's treatment and asked for the withdrawal of ventilator support, recognizing that this step would likely result in the patient's passing. It is important to note that euthanasia is not legal in our country. During the withdrawal of life-sustaining therapy, our primary concern is to alleviate distressing symptoms and ensure the patient's comfort. Among these 29 patients, 16 were female. Finally, 45 patients (18%) remained on ventilation, with 11 on non-invasive mechanical ventilation (NIV). All patients on NIV were discharged from the PICU and survived, while 34 patients were on invasive mechanical ventilation (IMV), with 2 still in the PICU receiving PMV.

The median age of patients requiring PMV was 2.3 (0.7, 6.8) years, with 57% of them being male. IMV was the predominant mode of mechanical ventilation at the time of diagnosis, accounting for 93.6% of cases (234/250). Additionally, 19% of patients (48/250) underwent tracheostomy. Among them, 19 were successfully weaned off ventilation, 10 died, while 19 out of 34 patients who remained on IMV underwent tracheostomy. The primary associated conditions of PMV were lower airway diseases (36%), central nervous system diseases (32%), and neuromuscular diseases (14%), as indicated in Table 1.

**Table 1:** Baseline characteristics of PMV patients in survival and death groups.

Characteristics	Survival group *N* = 160	Death group *N* = 90	*X/Z*	*P* value
Age, year	2.6 (0.6, 6.9)	2.1 (0.7, 6.7)	−0.058	.954
Male, *n* (%)	86 (53.8)	56 (62.2)	1.685	.194
Weight, kg	11 (7, 23)	11 (7, 18)	−0.1603	.873
BMI, kg/m^2^	15.4 (13.6, 17.4)	16.0 (13.7, 17.9)	−1.034	.301
Potential factors for death				
Associated conditions of PMV, *n* (%)			9.607	.142
Lower airway diseases	53 (33.1)	37 (41.1)		
Central nervous system diseases	54 (33.8)	27 (30.0)		
Neuromuscular diseases	26 (16.3)	8 (8.9)		
Upper airway diseases	10 (6.2)	3 (3.3)		
Cardiovascular diseases	5 (3.1)	4 (4.5)		
Genetic syndromes	3 (1.9)	0 (0)		
Other	9 (5.6)	11 (12.2)		
PELOD-2 score on the day of PMV diagnosis	4 (3, 5)	6 (4, 9)	−6.483	**<**.**001**
Respiratory				
Mode of ventilation, *n* (%)			4.097	.**043**
IMV	146 (91.2)	88 (97.8)		** **
NIV	14 (8.8)	2 (2.2)		** **
Tracheostomy, *n* (%)	38 (23.8)	10 (11.1)	5.931	.**015**
Timing of tracheostomy, day	27 (20, 38)	30 (13, 47)	−0.510	.610
MV duration, day	30 (21, 48)	22 (21, 41)	1.898	.058
VAP^a^, *n* (%)	51 (35.9)	30 (39.5)	0.268	.604
Central nervous system				
GCS on the day of PMV diagnosis	15 (7,15)	8 (4,15)	3.869	**<**.**001**
Nutrition				
Serum albumin on the day of PMV diagnosis, g/L	36.6 (32.5, 39.7)	36.3 (32.9, 40.3)	0.020	.984
Total calories intake on the day of PMV diagnosis, kcal/kg	56 (51, 64)	56 (42, 56)	2.968	.**003**
Protein deliveries on the day of PMV diagnosis, g/kg	1.3 (0.9, 2)	1.1 (0.8, 1.5)	2.346	.**019**
Use of antibiotics, *n* (%)	132 (82.5)	86 (92.2)	4.522	.**033**
Use of vasoactive agents, *n* (%)	18 (11.2)	39 (43.3)	33.684	**<**.**001**
Rehabilitation, *n* (%)	106 (66.2)	40 (44.4)	11.273	.**001**
PICU stay, day	44(33, 71)	40(29, 64)	1.577	.115

BMI, body mass index; PMV, prolonged mechanical ventilation; PELOD-2, pediatric logistic organ dysfunction-2; IMV, invasive mechanical ventilation; NIV, non-invasive mechanical ventilation; MV, mechanical ventilation; VAP, ventilator associated pneumonia; GCS, Glasgow coma scale; PICU, pediatric intensive care unit.

^a^ Data only available for 218 patients.

Bold values represent *P*-values less than 0.05, which are statistically significant.

### 3.2 Characteristics of PMV patients in survival and death groups

Table 1 presented the characteristics of PMV patient on the day of PMV diagnosis. The clinical features, including age, gender, weight, BMI, and the cause of PMV, were comparable between the groups at baseline. The median duration from intubation to tracheotomy was 27 days (range 21–40 days), and the median duration of mechanical ventilation was 29 days (range 21–46 days).

In comparison to the survival group (Table 1), the death group exhibited a higher PELOD-2 score (6 vs. 4, *P* = 0.000) and a lower Glasgow Coma Scale (GCS) score (8 vs. 15, *P* < .001) on the day of PMV diagnosis. Additionally, the use of antibiotics and vasoactive agents on the day of PMV diagnosis was more prevalent in the death group. Conversely, the survival group showed higher total calorie intake (*P* = .003) on the day of PMV diagnosis and a greater proportion of rehabilitation (*P* = .001) were noted in the survival group.

It can also be observed that there was a significant difference in the calorie and protein intake between the survival group and the death group at the time of PMV diagnosis. For total calorie intake, the median value for the survival group was 56 kcal/kg (range 51–64 kcal/kg), while for the death group, it was 56 kcal/kg (range 42–56 kcal/kg), with a *P*-value of 0.003, indicating a statistically significant difference. Similarly, for protein deliveries, the survival group had a median intake of 1.3 g/kg (range 0.9–2 g/kg), while the death group had a median intake of 1.1 g/kg (range 0.8–1.5 g/kg), with a *P*-value of 0.019, indicating a statistically significant difference. The survival group had a higher calorie and protein intake on the day of PMV diagnosis compared to the death group, and these differences were statistically significant.

### 3.3 Risk factors for death among PMV patients

We conducted a stepwise multiple logistic regression analysis, incorporating variables that exhibited statistical significance (*P* < 0.05) in Table 1. These variables included the Pediatric Logistic Organ Dysfunction-2 (PELOD-2) score, mode of ventilation, tracheostomy, Glasgow Coma Scale (GCS) at the time of prolonged mechanical ventilation (PMV) diagnosis, total calorie intake on the day of PMV diagnosis, protein delivery on the day of PMV diagnosis, use of antibiotics, use of vasoactive agents, and rehabilitation. The results of the analysis indicated that the utilization of vasoactive agents and an elevated PELOD-2 score on the day of PMV diagnosis were significantly associated with an increased risk of PMV death.

Specifically, the OR for vasoactive agent use was 2.86 (95% CI: 0.15–0.84; *P* = 0.018), indicating that the use of vasoactive agents was associated with a 2.86-fold increase in the odds of PMV death. Additionally, for the PELOD-2 score, the OR was 1.37 (95% CI: 1.17–1.61; *P* < .001), indicating that a one-unit increase in the PELOD-2 score was associated with a 1.37-fold increase in the odds of PMV death.

On the other hand, early rehabilitation intervention was negatively associated with the risk of PMV death (OR* *= 0.45; 95% CI: 0.22–0.93; *P* = 0.032), indicating that early rehabilitation intervention was associated with a 55% reduction in the odds of PMV death (Table 2).

**Table 2:** Stepwise logistic regression analysis for risk factors associated with death among PMV patients.

Risk factors	Odds ratio	95% CI	*P* value
Use of vasoactive agents	2.86	0.15–0.84	.**018**
PELOD-2 on the day of PMV diagnosis	1.37	1.17–1.61	**<**.**001**
Rehabilitation	0.45	0.22–0.93	.**032**

PELOD-2, pediatric logistic organ dysfunction-2*;* CI, confidence interval.

Bold values represent *P*-values less than 0.05, which are statistically significant.

### 3.4 Factors associated with unsuccessful weaning

Table 3 presented the division of surviving PMV patients into two groups based on weaning outcomes. Statistically significant differences were observed between the two groups in terms of gender, associated conditions of PMV, tracheostomy, duration of mechanical ventilation and length of stay in the PICU.

**Table 3:** Comparison of survival PMV patients’ characteristics according to wean outcome on 180 days of follow-up.

Characteristics	Successful weaning *N* = 115	Unsuccessful weaning *N* = 45	*X/Z*	*P* value
Age, year	2.6 (0.5, 7.0)	2.6 (1.3, 5.8)	−0.725	.468
Male, *n* (%)	56 (48.7)	30 (66.7)	4.202	.**040**
Weight, kg	11 (6, 22)	14 (9, 23)	−1.158	.247
BMI, kg/m^2^	15.4 (13.7, 17.5)	15.2 (13.3, 16.6)	0.972	.331
Associated conditions of PMV, *n* (%)			23.225	.**001**
Lower airway diseases	44 (38.3)	9 (20.0)		
Central nervous system diseases	40 (34.8)	14 (31.1)		
Neuromuscular diseases	9 (7.8)	17 (37.8)		
Upper airway diseases	9 (7.8)	1 (2.2)		
Cardiovascular diseases	4 (3.5)	1 (2.2)		
Genetic syndromes	2 (1.7)	1 (2.2)		
Other	7 (6.1)	2 (4.5)		
Respiratory				
Mode of ventilation, *n* (%)			0.340	.560
IMV	104 (90.4)	42 (93.3)		
NIV	11 (9.6)	3 (6.7)		
Tracheostomy, *n* (%)	19 (16.5)	19 (42.2)	11.797	.**001**
Timing of tracheotomy, day	21 (15, 30)	32 (21, 58)	−2.493	.**013**
Pre-PMV controlled ventilation duration, day	12 (1, 20)	13 (4, 21)	−1.173	.241
MV duration, day	26 (21, 36)	49 (28, 61)	−4.013	**<**.**001**
PaO_2_/FiO_2_ ratio	279 (191, 486)	301 (215, 463)	−0.265	.791
Lung infiltration area >1/4(CXR)	53 (46.1)	16 (35.6)	1.463	.227
Cardiovascular				
LVEF on the day of PMV diagnosis	65 (65, 65)	65 (65, 65)	−0.519	.604
Use of vasoactive agents, *n* (%)	14 (12.2)	4 (8.9)	0.350	.554
Central nervous system				
GCS on the day of PMV diagnosis	12 (7, 15)	15 (8, 15)	−0.352	.725
Infection				
Use of antibiotics, *n* (%)	95 (83.5)	36 (80.0)	0.271	.603
VAP^a^	39 (38.2)	12 (30.0)	0.847	.358
Nutrition				
Serum albumin on the day of PMV diagnosis, g/L	36.1 (32.1, 39.7)	37.8 (33.3, 40.4)	−1.237	.216
Total calories intake on the day of PMV diagnosis, kcal/kg	56 (50, 62)	56 (54, 65)	−0.220	.826
Protein deliveries on the day of PMV diagnosis, g/kg	1.4 (0.8, 2.1)	1.2 (0.9, 2.0)	−0.523	.601
Rehabilitation, *n* (%)	75 (65.2)	31 (68.9)	0.195	.659
PICU stay, day	42(32, 60)	61(42, 84)	−3.158	.**002**

BMI, body mass index; PMV, prolonged mechanical ventilation; PELOD-2, pediatric logistic organ dysfunction 2; MV, mechanical ventilation; PaO_2_, partial pressure of oxygen; FiO_2_, fraction of inspiration oxygen; CXR, chest x-ray; LVEF, left ventricular ejection fraction; GCS, Glasgow coma scale; VAP, ventilator associated pneumonia; PICU, pediatric intensive care unit.

^a^ Data only available for 124 patients.

Bold values represent *P*-values less than 0.05, which are statistically significant.

In the group with unsuccessful weaning, neuromuscular diseases were the most common cause of PMV (38%), followed by central nervous system diseases (31%), lower airway diseases (20%). Conversely, lower respiratory diseases were the predominant cause in the group with successful weaning (38%). Male patients and those who underwent tracheostomy were more prevalent in the unsuccessful weaning group. Among the patients experiencing weaning failure, 42% (19/45) underwent tracheostomy, with a median tracheostomy during of 32 (21, 58) days. Shorter duration of mechanical ventilation support (*P* < .001) and PICU stay (*P* = .002) were observed in the successful weaning group. The duration of controlled ventilation before PMV diagnosis was not found to be associated with weaning failure.

Table 4 displayed the result of the multiple logistic regression analysis for risk factors associated with failure to wean. The analysis included gender, associated conditions of PMV, and the timing of tracheostomy. As detailed in Table 3, statistical significance for these factors was set at a level of *P* < 0.05. Notably, tracheostomy and the length of PICU stay were excluded from the analysis, as they were not typically considered causative factors in clinical practice. The results of the analysis revealed that neither gender nor associated conditions of PMV were statistically significant. However, the timing of tracheostomy was found to have a significant effect, with an OR of 1.08 (95% CI: 1.01–1.16) and a *P*-value of 0.030. This suggest that there was a slight increase in the risk of weaning failure for patients who undergo tracheostomy later in their treatment course.

**Table 4:** Multiple logistic regression analysis for risk factors associated with weaning among survival PMV patients.

Risk factors	Odds ratio	95% *CI*	*P* value
Gender	0.33	0.07–1.58	.166
Associated conditions of PMV	0.62	0.29–1.33	.217
Timing of tracheotomy	1.08	1.01–1.16	.**030**

CI, confidence interval.

Bold values represent *P*-values less than 0.05, which are statistically significant.

## 4 Discussion

We conducted a multicenter prospective cohort study to report the incidence and 180-day outcomes of PMV patients in Chinese PICUs. Our study also revealed that the use of vasoactive agents and a higher PELOD-2 scores were associated with an increased risk of mortality in children receiving PMV children, while early rehabilitation intervention seemed to enhance the outcomes of these patients. To enhance the successful weaning process, it is advocated that early tracheostomy be considered for pediatric patients at a higher risk of non-extubation.

This study represented the first comprehensive analysis of the incidence of PMV in children undergoing mechanical ventilation in China, revealing a PMV incidence rate was 5.3%. Unfortunately, the short-term prognosis for these patients was not optimistic. Previous research has indicated high fatality rates associated with PMV, with a 1-year mortality rate of 59% in adults (19), a 27% fatality rate for children on HMV (20), and a 45% fatality rate for children with PMV in Taiwan (10). In our study, the fatality rate at 180 days of follow-up was 36%, primarily attributed to the severity of the underlying disease and challenges in weaning patients off ventilators.

Successful weaning from mechanical ventilation is crucial for patient outcomes. Various factors (21), including lung, brain, and heart function, as well as endocrine, nutrition, internal environment status, can influence the weaning process. Despite these complexities, our multivariable logistic regression analysis identified the timing of tracheostomy as an independent risk factor for weaning failure. Tracheostomy, in theory, may aid weaning by optimizing respiratory mechanics, secretion clearance, sedation requirements, and aspiration risk. Guideline recommend considering early tracheostomy in patients requiring PMV (22). However, there is no consensus on the optimal timing of tracheostomy in children, and in our study, most tracheostomies were performed after 3 weeks of intubation. This suggests that delayed tracheostomy may negatively impact ventilator weaning success. Recently, a large cohort study published in Pediatric Critical Care Medicine (PCCM) revealed that tracheostomy placement within 14 days of MV was associated with improved in-hospital outcomes (23). The rationale for early tracheostomy in children is centered on the potential to improve weaning outcomes, reduce complications, enhance patient comfort, and facilitate rehabilitation. However, careful consideration of the associated risks and individual patient factors is crucial for informed decision-making.

Identifying modifiable risk factors for mortality can facilitate the development of targeted clinical interventions to enhance the prognosis of children undergoing PMV. In our analysis of 180 days of follow-up data, we identified early rehabilitation intervention as a protective factor against death. This finding is in line with the growing recognition of the significance of early rehabilitation in intensive care medicine. Numerous clinical studies have underscored the crucial role of early rehabilitation in enhancing patient outcomes, facilitating ventilator weaning, and preventing complications. The integration of early rehabilitation strategies may offer substantial benefits in improving the overall prognosis of PMV children (24–27).

In terms of the associated conditions of PMV, the findings of this study align with the majority of previous research. The primary associated conditions identified were brain dysfunction, neuromuscular diseases, and chronic lung diseases. However, concerning the type of ventilation, our study revealed that IMV accounted for 94% of cases at the time of PMV diagnosis, a notably higher percentage compared to other studies (7, 28–30). Conversely, the utilization of non-invasive ventilation was significantly lower. In China, invasive ventilators remain the predominant choice, with non-invasive ventilators serving as supplementary equipment. The limited popularity of NIV can be attributed to factors such as a short time on the market, substantial time and cost investments required for medical education, and the need for continuous adjustments based on patient feedback, including sound levels and mask fit, which can be time-consuming. As a result, healthcare providers often opt to administer oxygen to milder cases and directly employ invasive ventilator for severe cases. Through the implementation of a sequential invasive-noninvasive mechanical ventilation weaning strategy and the establishment of a family mechanical ventilation management system in China, the perception and utilization of non-invasive ventilation are expected to strengthen over time. Healthcare professionals are likely to enhance their proficiency in utilizing non-invasive ventilation, leading to a potential increase in its adoption rate in the future.

Our study examined various factors contributing to weaning failure, including brain function (GCS), lung function (PaO_2_/FiO_2_ ratio, CXR), heart function (LVEF) and nutritional status (BMI, albumin, energy intake), however, no statistically significant results were obtained. This could be attributed to the complex nature of factors influencing weaning, where no single variable exerts a dominant influence (21, 31). Previous research by Jung-Rern Jiang et al. utilized the modified Burns Wean Assessment Program scores to predict successful weaning in the adult patients (32), incorporating 20 variables with a sensitivity and specificity of 81.4% and 82.1%, respectively. Comparable studies focusing on pediatric patients are currently lacking.

While our study represented the largest investigation into the incidence, outcomes, and prognostic factors of PMV in children in China, it was not without limitations. Firstly, PMV was a chronic condition and due to the recent establishment of the PMV cohort, the follow-up period in our study was relatively short. Our research team plans to conduct long-term follow-ups spanning 5–10 years. Secondly, advancements in ventilator weaning techniques, such as diaphragm function assessment (33, 34), ultrasound technology (35) and the NAVA weaning strategy (36) have shown promising results in recent years. Our study did not capture relevant data on these measures, leading to an incomplete analysis of weaning factors that warrants further exploration. Lastly, while our study primarily focused on ventilator weaning, the process of extubation also represents a crucial outcome that necessitates further investigation.

## 5 Conclusions

This study uncovered a 5.3% incidence of prolonged mechanical ventilation (PMV) among children in China requiring mechanical ventilation. A higher PELOD-2 score at the time of PMV diagnosis was linked to an increased mortality rate during PMV. Early rehabilitation was identified as a protective factor for the prognosis of patients undergoing PMV. In the context of weaning determinants, the timing of tracheostomy emerged as a significant risk factor. It is plausible that healthcare providers may have been inclined to perform tracheostomies earlier and with greater frequency in pediatric patients whom they deemed unlikely to be successfully extubated. This practice could potentially impact the weaning process and warrants further investigation to optimize clinical outcomes in this vulnerable population.

## Data Availability

The original contributions presented in the study are included in the article/Supplementary Material, further inquiries can be directed to the corresponding authors.

## References

[1] Maddux AB Pinto N Fink EL Hartman ME Nett S Biagas K Postdischarge outcome domains in pediatric critical care and the instruments used to evaluate them: a scoping review. Crit Care Med. (2020) 48:e1313–21. 10.1097/CCM.0000000000004595 33009099 PMC7708523

[2] Liu Y Wang Q Hu J Zhou F Liu C Li J Characteristics and risk factors of children requiring prolonged mechanical ventilation vs. non-prolonged mechanical ventilation in the PICU: a prospective single-center study. Front Pediatr. (2022) 10:830075. 10.3389/fped.2022.830075 35211431 PMC8861196

[3] Sahetya S Allgood S Gay PC Lechtzin N. Long-term mechanical ventilation. Clin Chest Med. (2016) 37:753–63. 10.1016/j.ccm.2016.07.014 27842754 PMC7273179

[4] Park M Jang H Sol IS Kim SY Kim YS Kim YH Pediatric home mechanical ventilation in Korea: the present situation and future strategy. J Korean Med Sci. (2019) 34:e268. 10.3346/jkms.2019.34.e268 31674158 PMC6823518

[5] Khirani S Amaddeo A Griffon L Lanzeray A Teng T Fauroux B. Follow-up and monitoring of children needing long term home ventilation. Front Pediatr. (2020) 8:330. 10.3389/fped.2020.00330 32656168 PMC7322995

[6] Leske V Guerdile MJ Gonzalez A Testoni F Aguerre V. Feasibility of a pediatric long-term home ventilation program in Argentina: 11 years’ experience. Pediatr Pulmonol. (2020) 55:780–7. 10.1002/ppul.24662 31977167

[7] Pavone M Verrillo E Onofri A Caggiano S Chiarini Testa MB Cutrera R. Characteristics and outcomes in children on long-term mechanical ventilation: the experience of a pediatric tertiary center in Rome. Ital J Pediatr. (2020) 46:12. 10.1186/s13052-020-0778-8 32005269 PMC6995086

[8] Manning JC Pinto NP Rennick JE Colville G Curley MAQ. Conceptualizing post intensive care syndrome in children-the PICS-p framework. Pediatr Crit Care Med. (2018) 19:298–300. 10.1097/PCC.0000000000001476 29406379

[9] Damuth E Mitchell JA Bartock JL Roberts BW Trzeciak S. Long-term survival of critically ill patients treated with prolonged mechanical ventilation: a systematic review and meta-analysis. Lancet Respir Med. (2015) 3:544–53. 10.1016/S2213-2600(15)00150-2 26003390

[10] Pai SC Kung PT Chou WY Kuo T Tsai WC. Survival and medical utilization of children and adolescents with prolonged ventilator-dependent and associated factors. PLoS One. (2017) 12(6):e017927. 10.1371/journal.pone.0179274 PMC5476277 28628663

[11] Trudzinski FC Neetz B Bornitz F Muller M Weis A Kronsteiner D Risk factors for prolonged mechanical ventilation and weaning failure: a systematic review. Respiration. (2022) 101:959–69. 10.1159/000525604 35977525 PMC9677859

[12] Zhang Z Tao J Cai X Huang L Liu C Ren H Clinical characteristics and outcomes of children with prolonged mechanical ventilation in PICUs in mainland China: a national survey. Pediatr Pulmonol. (2023) 58(5):1401–10. 10.1002/ppul.26332 36705329

[13] MacIntyre NR Epstein SK Carson S Scheinhorn D Christopher K Muldoon S Management of patients requiring prolonged mechanical ventilation: report of a NAMDRC consensus conference. Chest. (2005) 128:3937–54. 10.1378/chest.128.6.3937 16354866

[14] Sauthier M Rose L Jouvet P. Pediatric prolonged mechanical ventilation: considerations for definitional criteria. Respir Care. (2017) 62:49–53. 10.4187/respcare.04881 27879381

[15] G. Pediatric Acute Lung Injury Consensus Conference. Pediatric acute respiratory distress syndrome: consensus recommendations from the pediatric acute lung injury consensus conference. Pediatr Crit Care Med. (2015) 16:428–39. 10.1097/PCC.0000000000000350 25647235 PMC5253180

[16] Feng X Suyun Q. Interpretation of experts’ consensus on sedation and analgesia for children in pediatric intensive care unit of China (2018). Chin J Pediatr. (2019) 57(5):336–7. 10.3760/cma.j.issn.0578-1310.2019.05.004 31060124

[17] Ministry of Health of the People's Republic of China. Diagnostic criteria for nosocomial infection Chinese. Chin Med J. (2001) 81(5):314–20. 10.3760/j:issn:0376-2491.2001.05.027

[18] Yankov IV Shmilev TI. Ventilator-associated pneumonias in children (I)–diagnostic criteria, etiology and pathogenesis. Folia Med (Plovdiv). (2012) 54:5–11. 10.2478/v10153-011-0071-0 22908824

[19] Amaddeo A Frapin A Fauroux B. Long-term non-invasive ventilation in children. Lancet Respir Med. (2016) 4:999–1008. 10.1016/S2213-2600(16)30151-5 27423917

[20] Sterni LM Collaco JM Baker CD Carroll JL Sharma GD Brozek JL An official American thoracic society clinical practice guideline: pediatric chronic home invasive ventilation. Am J Respir Crit Care Med. (2016) 193:e16–35. 10.1164/rccm.201602-0276ST 27082538 PMC5439679

[21] Heunks LM van der Hoeven JG. Clinical review: the ABC of weaning failure–a structured approach. Crit Care. (2010) 14:245. 10.1186/cc9296 21143773 PMC3220047

[22] MacIntyre NR Cook DJ Ely EW Jr Epstein SK Fink JB Heffner JE Evidence-based guidelines for weaning and discontinuing ventilatory support: a collective task force facilitated by the American college of chest physicians; the American association for respiratory care; and the American college of critical care medicine. Chest. (2001) 120:375S–95. 10.1378/chest.120.6_suppl.375S 11742959

[23] Mehrotra P Thomas C Gerber LM Maresh A Nellis M. Timing of tracheostomy in critically ill infants and children with respiratory failure: a pediatric health information system study. Pediatr Crit Care Med. (2023) 24:e66–75. 10.1097/PCC.0000000000003120 36508241

[24] Gundogdu I Ozturk EA Umay E Karaahmet OZ Unlu E Cakci A. Implementation of a respiratory rehabilitation protocol: weaning from the ventilator and tracheostomy in difficult-to-wean patients with spinal cord injury. Disabil Rehabil. (2017) 39:1162–70. 10.1080/09638288.2016.1189607 27339104

[25] Balas MC Vasilevskis EE Burke WJ Boehm L Pun BT Olsen KM Critical care nurses’ role in implementing the “ABCDE bundle” into practice. Crit Care Nurse. (2012) 32:35–8. 40–7; quiz 48. 10.4037/ccn2012229 22467611 PMC3375171

[26] Anekwe DE Biswas S Bussieres A Spahija J. Early rehabilitation reduces the likelihood of developing intensive care unit-acquired weakness: a systematic review and meta-analysis. Physiotherapy. (2020) 107:1–10. 10.1016/j.physio.2019.12.004 32135387

[27] Bissett B Leditschke IA Green M Marzano V Collins S Van Haren F. Inspiratory muscle training for intensive care patients: a multidisciplinary practical guide for clinicians. Aust Crit Care. (2019) 32:249–55. 10.1016/j.aucc.2018.06.001 30007823

[28] Tsang YP To CY Tsui CK Leung SY Kwok KL Ng DK. Feasibility of long-term home noninvasive ventilation program in a general pediatric unit: 21 years’ experience in Hong Kong. Pediatr Pulmonol. (2021) 56:3349–57. 10.1002/ppul.25593 34339596

[29] Kim HI Cho JH Park SY Lee YS Chang Y Choi WI Home mechanical ventilation use in South Korea based on national health insurance service data. Respir Care. (2019) 64:528–35. 10.4187/respcare.06310 30584068

[30] McDougall CM Adderley RJ Wensley DF Seear MD. Long-term ventilation in children: longitudinal trends and outcomes. Arch Dis Child. (2013) 98:660–5. 10.1136/archdischild-2012-303062 23838128

[31] Wei X Day AG Ouellette-Kuntz H Heyland DK. The association between nutritional adequacy and long-term outcomes in critically ill patients requiring prolonged mechanical ventilation: a multicenter cohort study. Crit Care Med. (2015) 43:1569–79. 10.1097/CCM.0000000000001000 25855901

[32] Jiang JR Yen SY Chien JY Liu HC Wu YL Chen CH. Predicting weaning and extubation outcomes in long-term mechanically ventilated patients using the modified burns wean assessment program scores. Respirology. (2014) 19:576–82. 10.1111/resp.12266 24661343

[33] Hung TY Wu WL Kuo HC Liu SF Chang CL Chang HC Effect of abdominal weight training with and without cough machine assistance on lung function in the patients with prolonged mechanical ventilation: a randomized trial. Crit Care. (2022) 26:153. 10.1186/s13054-022-04012-1 35614518 PMC9131694

[34] Zhang Q Zhou J Zhu D Zhou S. Evaluation of the effect of high protein supply on diaphragm atrophy in critically ill patients receiving prolonged mechanical ventilation. Nutr Clin Pract. (2022) 37:402–12. 10.1002/ncp.10672 34101252

[35] Schepens T Fard S Goligher E. Assessing diaphragmatic function. Respir Care. (2020) 65:807–19. 10.4187/respcare.07410 32457172

[36] Vaporidi K. NAVA And PAV+ for lung and diaphragm protection. Curr Opin Crit Care. (2020) 26:41–6. 10.1097/MCC.0000000000000684 31738231

